# Smart Cutting Tools Used in the Processing of Aluminum Alloys

**DOI:** 10.3390/s22010028

**Published:** 2021-12-22

**Authors:** Dan Dobrotă, Sever-Gabriel Racz, Mihaela Oleksik, Ionela Rotaru, Mădălin Tomescu, Carmen Mihaela Simion

**Affiliations:** Faculty of Engineering, University Lucian Blaga of Sibiu, Str. Emil Cioran Nr. 4, 550025 Sibiu, Romania; gabriel.racz@ulbsibiu.ro (S.-G.R.); mihaela.oleksik@ulbsibiu.ro (M.O.); ionela.rotaru@ulbsibiu.ro (I.R.); madalin.tomescu@ulbsibiu.ro (M.T.); carmen.simion@ulbsibiu.ro (C.M.S.)

**Keywords:** smart tools, vibration analysis, aluminum alloys, cutting, FEM analysis, roughness

## Abstract

The processing of aluminum alloys in optimal conditions is a problem that has not yet been fully resolved. The research carried out so far has proposed various intelligent tools, but which cannot be used in the presence of cooling-lubricating fluids. The objective of the research carried out in the paper was to design intelligent tools that would allow a control of the vibrations of the tool tip and to determine a better roughness of the processed surfaces. The designed intelligent tools can be used successfully in the processing of aluminum alloys, not being sensitive to coolants-lubricants. In the research, the processing by longitudinal turning of a semi-finished product with a diameter Ø = 55 mm of aluminum alloy A2024-T3510 was considered. Two constructive variants of smart tools were designed, realized, and used, and the obtained results were compared with those registered for the tools in the classic constructive variant. The analysis of vibrations that occur during the cutting process was performed using the following methods: Fast Fourier Transform (FFT); Short-Time Fourier-Transformation (STFT); the analysis of signal of vibrations. A vibration analysis was also performed by modeling using the Finite Element Method (FEM). In the last part of the research, an analysis of the roughness of the processed surfaces, was carried out and a series of diagrams were drawn regarding curved profiles; filtered profiles; Abbott–Firestone curve. Research has shown that the use of smart tools in the proposed construction variants is a solution that can be used in very good conditions for processing aluminum alloys, in the presence of cooling-lubrication fluids.

## 1. Introduction

Machining of aluminum alloys presents a number of technical problems, especially if it is desired to be processed without coolants. Thus, the processing of aluminum alloys without the use of cooling-lubrication fluids generates the appearance of adhesions on the tool surface, determined by the low melting point and high ductility of these materials. Therefore, at present, a series of tools have been designed and used for processing aluminum alloys that have taken into account various materials and geometries for tools [1].

The geometry of the tools used in the processing of aluminum alloys was made based on different shapes of the seating and clearing surfaces, respectively. All this research has allowed an increase in the performance of these types of tools, but still, no tools have been made that have an optimal functional geometry. In order to be able to improve the cutting conditions of aluminum alloys, a series of intelligent tools have been proposed, but which present a series of difficulties of use in the conditions in which the cutting process takes place in the presence of cooling-lubrication fluids [2,3].

The use of intelligent tools in intelligent processing systems can have an extraordinary impact on the processing technologies of parts that require very good dimensional accuracy and very low roughness. In order to ensure high-precision machining processes, the use of intelligent cutting tools is important to cope with dynamic machining and large variations in conditions that may occur during machining processes [4].

Different methods can be used to analyze the evolution of cutting tools, the most commonly used being acoustic emission. Thus, using EA, two wear parameters (maximum wear land and crater wear width) can be set. Additionally, the use of EA allows establishing the moment when to change tools in unattended production systems. A main drawback in applying AE techniques for tool wear monitoring is represented by the dependence of the response on cutting conditions, especially in the case of realistic cutting operations [5].

Cutting machining processes are generally accompanied by variations in cutting forces caused by an improper tool functional geometry that can have a direct influence on machining results. The lack of an optimal functional geometry of the cutting tool can cause its rapid wear with effects on the increase of forces and vibrations when cutting. In order to avoid such effects, it is necessary to identify the best constructive variants of tools that allow a control of the dynamics of the machining processes. In order to control the dynamic aspects of a cutting process, a number of systems have been developed at present by various researchers, but which have many limitations in practical applications due to the fact that they do not always have the best geometry and, at the same time, they can also influence the rigidity of the technological system. Additionally, the use of dynamometers involves, in general, a dry cutting medium, which is not indicated, especially in the case of aluminum alloys. Under these conditions, the use of dynamometer-based systems to control cutting processes is not indicated, especially in the case of aluminum alloy processing [6,7].

In addition, at present, various sensors have been achieved that allow the monitoring of cutting processes. Thus, a series of sensors have been developed that allow temperature monitoring during cutting processes, but also these sensors have not allowed to obtain the best results in practice due to the fact that temperature measurements during cutting are not of best accuracy and, thus, a series of errors may occur [8,9,10].

In various research, several types of smart tools have been developed based on measuring the cutting force; temperature monitoring during cutting processes; automatic positioning of the tools in relation to the surface of the workpieces. All these types of tools have made it possible to achieve certain results in machining processes, but all these tool systems need to be perfected to reach new Industry 4.0-specific performance [11].

Cutting with cutting tools that cannot ensure an optimal functional geometry can cause a worsening of the cutting conditions in the sense of increasing the frictional forces on the clearance face, respectively, the placement of the tool. All these increases in force values cause excessive wear of the cutting tools due to the intensification of the phenomena of adhesion, abrasion, adhesion, and diffusion. These phenomena are very sensitive to temperature values and can be even more intense especially in the case of processing aluminum alloys [12].

Temperature control in the cutting process by using different types of coolants is not always a solution due to the fact that it can intensify the process of environmental pollution with effects on human health, but also on the process of contamination of parts surfaces. [13,14].

All these problems specified above can be solved by using a smart tool that allows to ensure an optimal functional geometry at all times, thus avoiding all thermal, dynamic effects and, implicitly, those of reducing the precision and quality of machined parts. These effects can be more evident especially in the case of processing parts made of hard-to-process alloys such as aluminum alloys. At present, the cutting tools used for the machining of aluminum alloys have a rigid construction that does not allow to ensure an optimal functional geometry nor the control of the dynamic phenomena that accompany the machining processes. Under these conditions, at present, the best technical conditions are not ensured in order to be able to achieve an optimal processing, from a technical and economic point of view, of aluminum alloys [15,16].

The vibration phenomenon is an undesirable phenomenon in machining processes, as it results in a reduction in tool life, surface quality, and machining productivity. A widely investigated method of vibration control is the attachment of various dampers in the structure of the system used in processing [17]. For example, the most used was the regulated mass damper (TMD) in the practical control of vibrations which can be linear or nonlinear [18,19,20,21,22]. The best TMDs have proven to be those with nonlinear action because they allow a reduction of vibrations especially in turning operations. Theoretical and practical analyses have shown that properly designed and adjusted nonlinear TMD can provide high performance for reducing processing vibrations. A 150–180% reduction in vibration amplitude can be achieved using a properly adjusted nonlinear TMD compared to a machining system without a shock absorber [23,24].

The analysis of research in the field, carried out to date, demonstrates the need to design, manufacture, and use intelligent cutting tools for processing aluminum alloys that allow vibration damping but also have an optimal functional geometry. This type of tool can also be used successfully when the cutting process is carried out under the conditions of using cooling-lubrication fluids. At the same time, the use of intelligent cutting tools allows an improvement of cutting conditions by controlling dynamic phenomena, namely of the vibrations, reducing the amount of energy consumed and improving the quality and accuracy of processed surfaces. Thus, the research carried out took into account the operation of processing by longitudinal turning of parts made of aluminum alloys with the use of a smart tool to ensure the best conditions for the development of processing. The main objective of the research was to make a smart tool to reduce vibrations that occur during machining, but also to ensure optimal functional geometry for the cutting tool throughout the process of machining aluminum alloys. The paper is structured in the following sections: materials and methods, results and discussions, conclusions.

## 2. Materials and Methods

### 2.1. Materials Used in Research

For the experimental research, cylindrical specimens with diameter Ø = 55 mm made of aluminum alloy A2024-T351: EN485 were used. The choice for the research of this aluminum alloy was made considering the good processing capacity of its cutting and the wide field of use (aircraft fittings, gears and shafts, bolts, clock parts, computer parts, couplings, fuse parts, hydraulic valve bodies, missile parts, ammunition, nuts, pistons, rectifier parts, worm gears, fastening devices, veterinary and orthopedic equipment, structures).

Regarding the chemical composition of the aluminum alloy A2024-T351, it is shown in Table 1, and the mechanical properties are shown in Table 2.

### 2.2. Equipment and Tools Used in the Processing by Cutting

**Figure 1:**
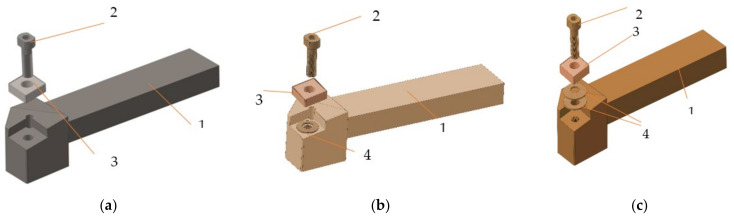
Knives for frontal turning: (**a**) in the classic version (T01); (**b**) with improved constructive form with a spring washer (T02); (**c**) with improved constructive form with two spring washers (T03); 1—knife body; 2—screw fixing; 3—removable plate; 4—spring washer.

For the experimental research, a technological equipment such as a lathe Victor VT 15 PLUS with numerical control with FANUC control was used, provided by FANUC Automation Romania S.R.L., Cluj, Romania. The research carried out took into account the processing of an aluminum alloy and the parameters indicated to be used in the processing of this material [10], must comply with certain conditions. Thus, the parameters were established: rotational speed *n* = 800 rpm, machining depth a_p_ = 1 mm, longitudinal feed f = 0.22 mm/rev. A longitudinal turning tool was used in the machining (Figure 1). It was constructively modified, starting from a standardized tool, provided by Seco Tools, Brașov, Romania. The constructive geometry of the tool used is characterized by the following geometric parameters: rake angle γ = 8°, clearance angle α = 6°, edge angle χ_r_ = 90°, angle edge inclination λ_s_ = 8°.

**Figure 2:**
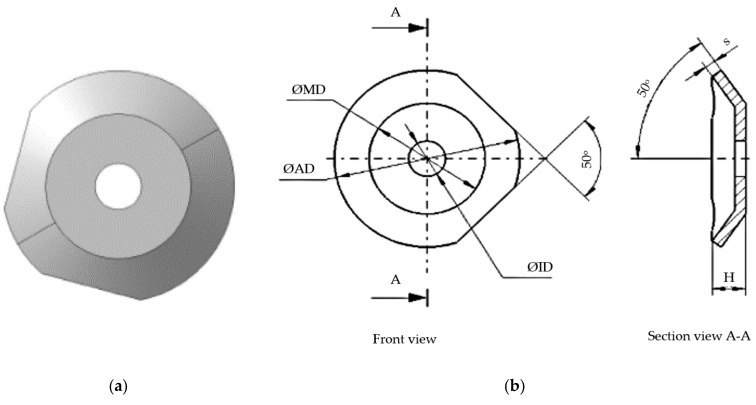
Constructively modified spring washer: (**a**) image; (**b**) dimensional elements (ØID = 5 mm; ØAD = 10 mm; ØMD = 7.5 mm H = 0.9 mm; s = 0.40 mm).

The process of longitudinal turning is quite complex, especially in the case of parts made of aluminum alloys, and this is determined by the physical and mechanical properties of these alloys and the functional geometry of the tools that change during machining. Thus, during machining, changes may occur in the functional angles of the cutting tool with effects on the conditions under which the cutting machining processes take place. For this reason, three types of tools were tested in the research: tool in the classic construction version T01 (Figure 1a), cutting tool T02 (Figure 1b), characterized so that a spring washer was inserted under the cutting plate (Figure 2), cutting tool T03 (Figure 1c), characterized in that a package of two spring washers was inserted under the cutting plate. By making these types of cutting tools (T02, T03), the aim was to create an intelligent tool with an optimal functional geometry.

Vibration control of the tool tip is of great importance for predicting the topography of the machined surface and active vibration control. The use of the intelligent tool in the constructive variants, presented in Figure 1b, respectively, Figure 1c, allows the control of the impact between the tool tip and the material to be processed. This impact control is possible due to the presence of a spring washer. Additionally, the modification of the spring washer, Figure 2, shape allows the takeover and control of vibrations. This is possible due to the fact that the washer is arranged on the tool body with the unprocessed part facing its tip. This spring washer arrangement was used because the greatest forces appear at the top of the tool.

As for the spring washer, disc spring, Figure 2, which corresponds to DIN 2093 B standard, steel A2 1.4305, and is produced by Vinsco Spring Limited, Changzhou, China, it was further processed by cutting, Figure 2a, so as to ensure an optimal value of the elastic system created on the entire active part of the cutting tool. 

### 2.3. Analysis of the Evolution of the Functional Geometry of the Tool When Turning with Longitudinal Advance

**Figure 3:**
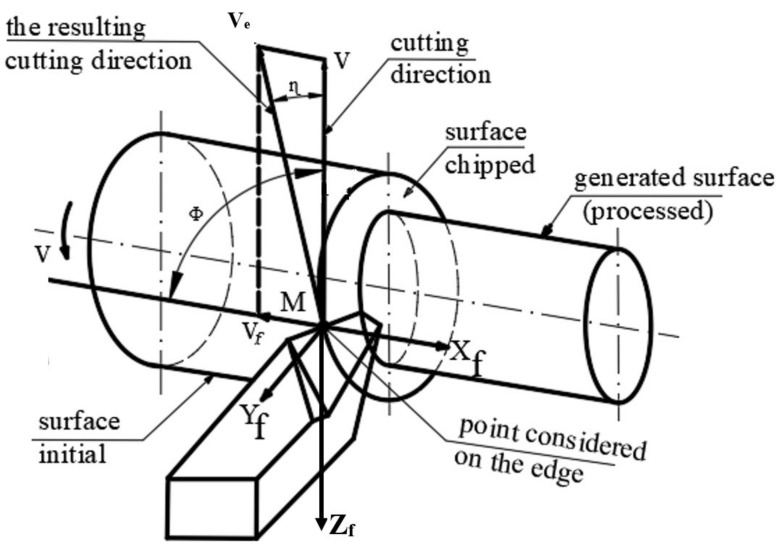
Kinematics of processing by turning with longitudinal feed.

The machining process by turning with a longitudinal advance of a part was carried out according to the kinematics shown in Figure 3. In the case of longitudinal turning, the difference between constructive and effective parameters is given by the variation of the direction of the effective speed (*V_e_*) compared to the main speed (*V*) taking into account the size of the feed rate (*V_f_*). With the help of the information presented in Figure 3, it is possible to determine the effective cutting speed, *V_e_*, which makes the angle *η* with the main cutting speed, *V*. Thus, the value of the angle *η* (angle of the main cutting direction) is determined from the speed triangle with equation:

(1) $$tg\eta =\frac{v_{f}}{v_{e}}=\frac{n\cdot f}{\pi \cdot D_{M}\cdot n}=\frac{f}{\pi \cdot D_{M}}$$

where *f* is the value of the working feed in mm/rev: *n*—part speed; *D_M_*—the diameter of the current point on the edge where the analysis is undertaken.

During the cutting process, the constructive geometry of the cutting tool will change into a functional geometry. The geometric changes produced refer to the clearance angle (*α*) and to the rake angle (*γ*). Thus, the functional geometry of the tool will be characterized by the effective seating angle (*α_Fe_*), respectively, the effective rake angle (*γ_Fe_*). Under these conditions, the angles that characterize the functional geometry of the tool can be expressed with the equations:

(2) $$\begin{array}{l} \gamma_{Fe}=\gamma +\eta \\ \alpha_{Fe}=\alpha -\eta \end{array}$$

Additionally, by replacing the angle *η*, the calculation equations of the functional geometry of the tool are obtained:

(3) $$\gamma_{Fe}=\gamma +arctg\frac{f}{\pi \cdot D_{M}}\;\alpha_{Fe}=\alpha +arctg\frac{f}{\pi \cdot D_{M}}$$

From Equations (2) and (3) it is observed that there may be differences between the constructive and the functional geometry, but only for the values established for the cross section of the cutting tool. Thus, the value of the clearance angle increases, which, under certain conditions, may be favorable, but the value of the setting angle decreases and there may be a danger of being too small or even negative, which would make the cutting operation no longer carried out under normal conditions. If the functional geometry of the cutting tool is analyzed in a longitudinal section, then the values of the corresponding angles can be determined with the equations:

(4) $$\begin{array}{l} \gamma_{Fe}=\gamma +arctg\frac{f}{\pi \cdot D_{M}}\sin\chi_{r}\\ \alpha_{Fe}=\alpha -arctg\frac{f}{\pi \cdot D_{M}}\sin\chi_{r} \end{array}$$

Thus, it is observed from Equation (4) that the functional geometry of the cutting tool, in the longitudinal section, also depends on the diameter of the *D_M_* analysis point, and this can determine the obtaining of a variable roughness depending on the diameter to be processed. In these conditions, it is necessary to identify the technical possibilities to obtain an optimal functional geometry, and this is possible by using intelligent cutting tools during processing, which adapt their geometry according to the processing conditions.

### 2.4. Measurement of Vibrations That Occur during the Cutting Process 

The cutting processes are always accompanied by vibration phenomena determined by their dynamic nature. Thus, during the machining operations a whole series of vibrations can appear which can be grouped into forced vibrations; autovibrations (self-excited vibrations); relaxation vibes. As the main objective of the research was to make a smart cutting tool, the aim was to find a technical solution to reduce the self-vibrations that accompany the cutting processes. Regarding the phenomenon of self-vibrations during cutting, they can be determined taking into account several theories/hypotheses [25]:

− Taylor’s hypothesis—the variation of the cutting forces, during the chip formation process, can determine the appearance of autovibrations;
− Kashirin’s theory—the adoption of an inappropriate geometry for the tool release face can cause the appearance of high frictional forces on the tool release face, which has effects on the autovibration phenomenon;
− Sokolcvsky’s theory—autovibrations are introduced by a variation of the geometry of the cutting tool during surface processing;
− Harnis and Grig’s theory—the continuous variation of the cutting depth can determine different values of the total force that appear in the cutting process, and this can determine the occurrence of autovibrations.

Based on the mentioned theories regarding the occurrence of self-vibrations and considering the main objective of the research, it can be appreciated that it is very important to ensure an optimal functional geometry for the cutting tool throughout the cutting process to reduce the autovibrations explained by Sokolcvsky’s theory and Kashirin’s theory.

Since the largest variation of the forces that appear during turning, determined by the functional geometry of the tools, is manifested in the Z and X directions, it was decided that in the research the vibrations should be measured in the two directions. The vibration measurement was performed considering the machining processes that involved the use of tools T01, T02, and T03 on the Y and Z directions.

**Figure 4:**
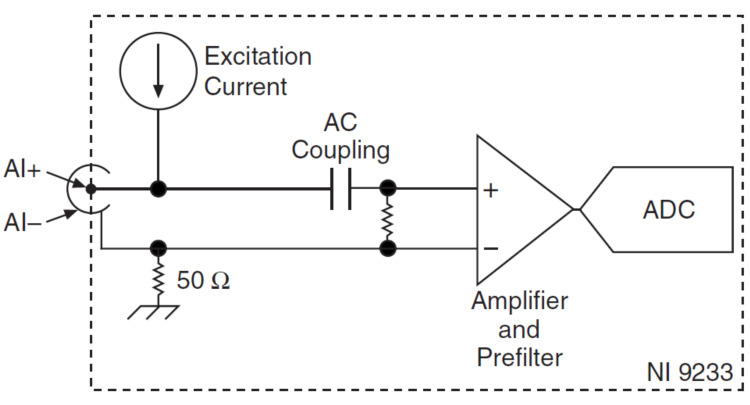
Scheme of a channel of the acquisition board NI USB-9233.

The research considered both the acquisition and processing of vibrations that occur during the machining processes that involved the use of the three types of tools. For this purpose, an acquisition board, NI USB-9233, was used, the structure of which is shown in Figure 4.

The data collection device used was the NI USB-9233. It has analog input channels and allows connection to IEPE sensors. As for the analog input channels of the acquisition board, they are connected to the chassis ground through a 50 Ω resistor. For the correct operation of the acquisition board, in order to minimize ground noise, it is necessary that it be connected to the ground. The acquisition board is also designed to be protected from surges. During operation the acquisition board provides an IEPE excitation current for each input signal. In order to obtain good conditions for taking over the information, the signal of the acquisition board is coupled to alternating current, buffered, and conditioned. In the next step, the received signals are sampled by a 24-bit Delta-Sigma ADC. Proper operation of the acquisition board also requires permanent activation of excitation current and AC coupling are always enabled.

**Figure 5:**
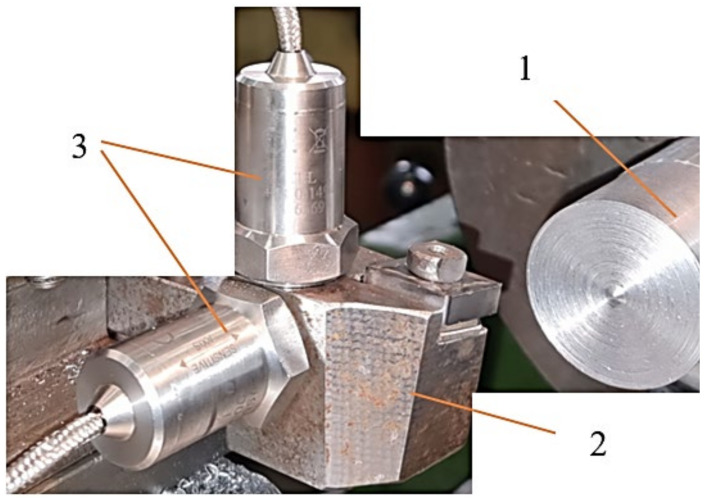
The arrangement of accelerometers: 1—workpiece; 2—tool; 3—accelerometers.

The measurement of vibrations in the two directions, Z and Y, respectively, involved mounting on the tool body two accelerometers arranged at a distance of 25 mm from the tool tip, Figure 5. A virtual tool, designed by the authors using the LabView program, was used to process the transmitted signal. The signals related to the measured and stored vibrations were subsequently processed and the Fast Fourier Transform (FFT) and Spectrogram were obtained in the Matlab program.

The two accelerometers used to measure the vibrations that occur during the cutting process were of the Monitran MTN/1100C type with a standard sensitivity of 100 mV/g and a frequency response from 2 Hz to 20 kHz. These two accelerometers were attached to the tool body and connected to the NI USB-9233 system.

### 2.5. Analysis of the Own Modes of Vibrations Using FEM

In order to confirm the values obtained for vibrations during experimental research, an analysis of its own vibration mode was performed using the Finite Element Method (FEM). The results obtained by FEM allow the comparison with similar results generated by other alternative methods and/or techniques including approximate and exact analytical solutions.

The use of FEM allows the establishment of natural frequencies for all three analyzed tools. The authors of the study performed the modal analysis for all tool variants (T01, T02, and T03) but in the paper the results are presented only for the T02 variant because it later proved to be the variant with the best results during the experimental research. To avoid the possibility that the part rotation frequency coincides with one of the natural frequencies of the tool, a modal analysis was performed based on finite element method. The modal analysis represents a possibility to study the behavior of the elastic systems through which the determination of the own frequencies of the different parts is aimed, the highlighting of some weak points of them, as well as the determination of the deformation tendencies in the dynamic field.

This type of analysis falls into the type of time-dependent analyses. Time-dependent problems can be reduced to a system of differential equations in which all matrices are symmetric. Equations can be linear or nonlinear if the stiffness matrices are dependent on the nonlinear properties of the materials or if large deformations occur.

Depending on the form of variation in time of the applied tasks, the answer of the structure is different, respectively, the form of the differential equation of motion changes. Thus, the following can be differentiated:

− determination of the response to free vibrations [f = 0]—modal analysis;
− determination of the excitation response with periodic forces [f(t)—Periodic]—harmonic analysis;
− determination of the excitation response in transient regime [f(t)—Transient]—analysis in transient regime.

**Figure 6:**
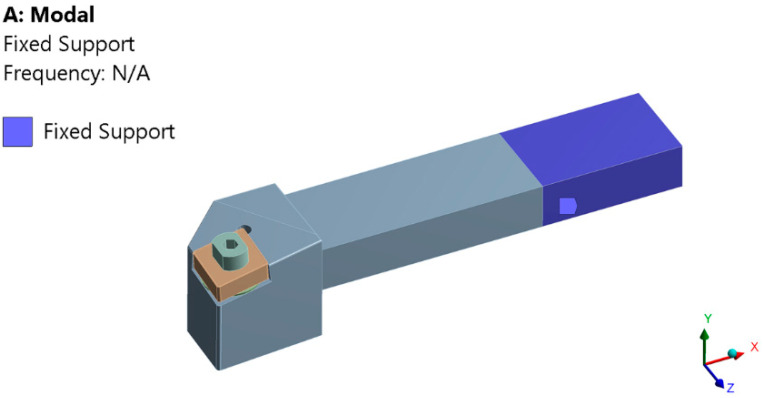
The constraints imposed on the T02 tool to perform the analysis of its own vibration modes.

In the first two cases, the initial conditions of the system do not matter and only a general solution is sought. The modal analysis performed in this case was a linear analysis, and as a method of extracting the eigenmodes the subspace iteration method using the generalized Jacobi algorithm was used. The method is preferred due to the precision of the results, because it works with whole stiffness and mass matrices, thus avoiding the need to choose degrees of freedom of the master type by the user. To perform the analysis, the tool was modeled in the Solidworks program, the model made using the STEP transfer standard was imported and it was discretized (meshed). A series of constraints were applied to the discretized model (a recess on the real area of the knife grip in the knife holder), Figure 6, and the analysis mode was run. It should be noted that no loads (cutting forces) were applied because the loads do not influence the determination of natural frequencies.

The geometric pattern imported from the CAD program was discretized, requiring a recess on all blue surfaces in Figure 6, these delimit the area of the knife body that is fixed in the knife holder. This area actually determines the area of the knife that remains free. For a highest possible accuracy of the results, each component part of the tool (knife body, spring washer, plate, screw) was discretized separately, making a combination of discretized areas with parallelepiped-shaped elements (on the fixing area) and tetrahedral elements (in the rest of the model). The minimum size of the finite element side was 0.0137 mm, so that the finite element network does not contain degenerate elements. Material properties were then assigned to each part and, since their own modes (natural frequencies) depend only on the body mass, its geometric shape and the mode of constraint, no loads were applied. The results obtained, shown in Figure 10 were focused on determining the natural frequencies and vibration trends of the knife, corresponding to these frequencies.

### 2.6. Measurement of the Roughness of the Processed Surfaces

The quality of the surfaces processed by cutting can be appreciated by their roughness. It should be noted that the roughness of the machined surfaces is influenced by many factors but in the research the focus was on the influence of the functional geometry of the tool and, implicitly, on the vibrations that occur during the machining process. Regarding the geometric parameters that most influence the roughness of the machined surfaces, these are the clearance angles and the laying angles. Under these conditions, during the research, the realization of a smart tool that allows to ensure an optimal functional geometry was a solution for obtaining a roughness as low as possible. In order to determine the influence of the tool geometry on the surface roughness, the functional geometry of the cutting tool was taken into account and thus, the dependence of the roughness Ra on the functional rake angle was established (*γ_Fe_*), Equation (5), respectively the functional placement angle (*α_Fe_*):

(5) $$R_{a}=0.25\cdot \frac{C_{v}\cdot K_{v}}{T^{xv}\cdot v_{f}}\cdot tg(\gamma_{Fe}-\gamma )$$

(6) $$R_{a}=0.25\cdot \frac{C_{v}\cdot K_{v}}{T^{xv}\cdot v_{f}}\cdot tg(\alpha -\alpha_{FE})$$

where: *C_v_*; *K_v_*; *x_v_* is the coefficients; *T*—tool durability; *V_f_*—feed rate.

From the analysis of Equations (5) and (6) it is observed that the roughness of the processed surface depends very much on the functional rake angle (*γ_Fe_*), respectively, the functional clearance angle (*α_Fe_*). Thus, by making the two variants of smart tools T02 and T03, respectively, it was desired to ensure a functional geometry for the cutting tool so that the roughness of the machined surface is maintained at the optimal values imposed for the respective part.

The measurement of the roughness of the surfaces processed by longitudinal turning was performed using an ST1 roughness meter provided by Hoffmann Industrial Tools S.R.L., Bucharest, Romania. All results obtained from roughness measurement were processed by MINITAB statistical software.

## 3. Results and Discussion 

The experimental research was performed considering the use of the three types of cutting tools (T01, T02, T03). Additionally, 10 samples were processed under the same conditions using the three tools. The processing of 10 samples was required to obtain an adequacy for the experimental results obtained. In the first stage of the research, the vibrations that occur during the cutting process were measured and all three types of cutting tools were taken into account. The part of the results regarding the vibrations was completed with the analysis of the own modes of vibrations in order to be able to make a comparison between the results obtained experimentally and those obtained by modeling.

In the last stage of the research, an analysis of the roughness of the processed surfaces was performed and, thus, it was possible to establish a correlation between the vibration parameters and the parameters that characterize the surface roughness of the parts. The analysis of the correlation between vibrations and roughness was performed using the STATISTICA software.

### 3.1. Analysis of Vibrations That Occur during the Cutting Process

Mechanical systems of the type used in research (cutting tool, machine tool part) are designed so that representative vibrations occur only at low frequencies specific to the range 10–1000 Hz [26,27]. Due to the fact that modern technological equipment that is used in cutting processes allows the use of very high speeds, the analysis of vibration frequency was extended to 10,000 Hz. In order to analyze the vibrations that accompany the cutting processes, it is necessary, first of all, an analysis of their amplitude. This characteristic of vibrations describes their severity and can be studied using various methods. For the analysis of the vibration amplitude the most indicated methods are those that establish the relationship between the peak-to-peak level, the peak level, and the average level of a sinewave. The most important relationship that can be used in the analysis of vibration amplitude is the peak-to-peak level because it helps to establish the maximum amplitude of the wave that occurs in case of a maximum load. As certain short-wave shocks can occur during the cutting process, the value of the peak-to-peak level can be used to determine their levels. This method is especially valuable for indicating the level of short-term shocks, etc. The average rectified value (RMS) can also be used in the practice of vibration analysis, because this value provides us with information on how the vibrations occurred during a cutting process. However, the RMS value is seldom used in vibration analysis because it has a limited interest in providing information about the vibrations that occur at a given time. In some cases, the value of RMS may be relevant for the measure of amplitude, as it shows the evolution of the wave over time and allows the establishment of a value for amplitude given the link between the energy content and the destructive capabilities of vibration.

**Figure 7:**
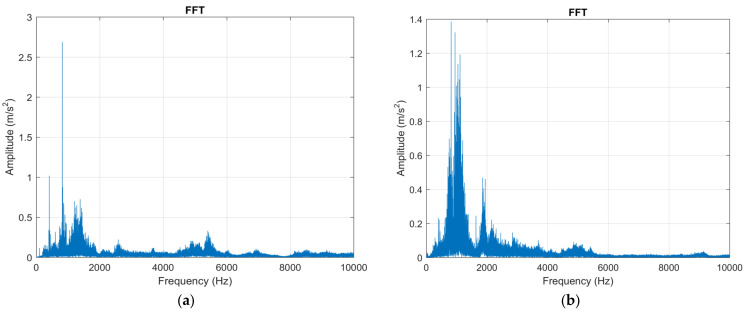

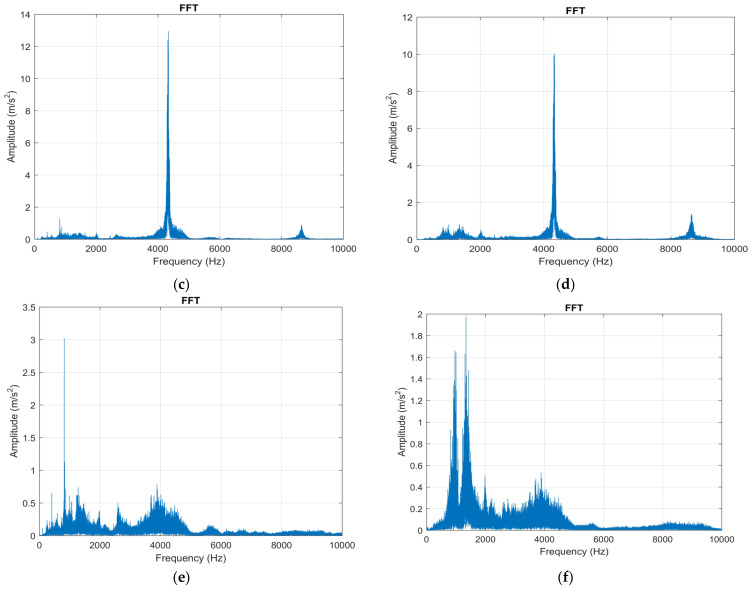
Vibration analysis by applying FFT: (**a**) in the Z direction in the case of machining with a cutting tool T01; (**b**) in the X direction in the case of machining with a cutting tool T01; (**c**) in the Z direction in the case of machining with a cutting tool T02; (**d**) in the X direction in the case of machining with a T02 cutting tool; (**e**) in the Z direction in the case of machining with a T03 cutting tool; (**f**) in the X direction in the case of machining with a T03 cutting tool.

The experimental research carried out aimed, in the first stage, to analyze the vibrations that occur during the machining of aluminum alloy using the three types of cutting tools. A first method used to analyze vibrations was to use Fast Fourier Transform (FFT) which is a method of analysis that can provide information about the vibration waveform. Vibration-specific waveforms are characterized by high complexity, and to perform an analysis of them the FFT method can be used allowing the decomposition of waveforms into a series of discrete sine waves. Thus, the FFT analysis was performed for the machining operations that involved the use of the three types of cutting tools (T01, T02, T03). The vibrations were analyzed in the two directions (X, Z), Figure 3, and the experimental results obtained for the analysis of vibrations using FFT are presented in Figure 7.

As it was specified in the case of the technological system consisting of the tool, part, device, machining machine, it is recommended that the vibration analysis be performed in the frequency range 10–10,000 Hz and it was made for a frequency range of up to 10,000 Hz. Vibration analysis using FFT for the situation in which all three types of cutting tools were tested allowed the issuance of the following conclusions:

− in the Z direction in the case of using the cutting tool T01 amplitude of vibrations has a maximum value of approximately 2.7 m/s^2^, and in the case of using the cutting tool T02 the amplitude of vibrations has a maximum value of approximately 1 m/s^2^. Thus, it was observed that when using the T02 cutting tool, a considerable decrease in vibration amplitude was obtained by 2.7 times. This demonstrates that the use of smart tools T02 allows a considerable decrease in the amplitude of vibrations which can be explained by the possibility of ensuring an optimal functional geometry for the cutting tool and the considerable reduction of frictional forces. After testing the T02 tool, the T03 tool was used in the research, which involved placing two spring washers under the removable plate. The FFT analysis performed when using this tool showed that the maximum amplitude of vibrations was 3 m/s^2^. Thus, the amplitude of vibrations when using this cutting tool is about 10% higher than when the cutting tool T01 was used and three times larger than when the cutting tool T02 was used. This demonstrates that, although in the case of the T03 tool an attempt was made to create a smart tool that would allow the adjustment of the functional geometry of the tool in a larger area, the rigidity of the cutting tool was greatly reduced. Under these conditions, in the case of tool T03, although a substantial reduction of the frictional forces can be obtained on the clearing and laying faces of the tool, nevertheless, this reduction of the frictional forces does not compensate for the decrease of the rigidity of the cutting tool. As a result, a correlation is required between the parameters of the spring system arranged under the removable plate, the cutting forces, the friction forces, and the rigidity of the cutting tool;
− the analysis of the amplitude of vibrations on the X direction in the case of using the cutting tool T01 showed a value of 1.4 m/s^2^. This lower value of the X-direction vibration amplitude compared to the Z-direction vibration amplitude is explained by the fact that the X-direction cutting forces are about half the Z-direction cutting forces. When using smart tool T02 in the X direction it was found that the amplitude of vibrations was about 0.03 m/s^2^. This demonstrates a considerable reduction in vibration amplitude and in the X direction when using the T02 smart tool. The amplitude of vibrations in the case of using the smart tool T03 was about 1.7 m/s^2^ which shows that even in the X direction the use of a tool that does not have a proper rigidity can cause an amplification of the vibration phenomenon with negative effects on the conditions in which the machining process is carried out.

Results obtained from the FFT analysis demonstrate that the use of a smart tool such as the T02 tool can reduce the vibrations that occur during the cutting process. However, in order to design a smart tool, the rigidity of the technological system must also be taken into account. Thus, if an unsuitable spring system is used under the removable plate, it may cause an accentuation of the vibration phenomenon with negative effects on the conditions under which the cutting process takes place [28].

**Figure 8:**
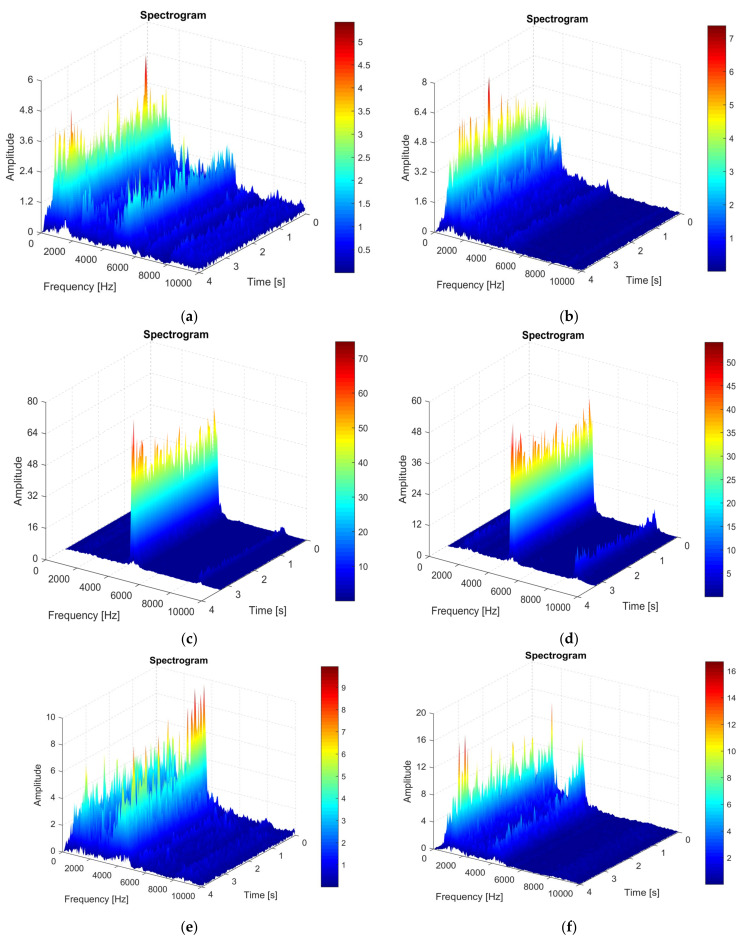
Vibration spectrogram recorded during machining: (**a**) in the Z direction in the case of machining with the cutting tool T01; (**b**) in the X direction in case of machining with the cutting tool T01; (**c**) in the Z direction in case of machining with the cutting tool T02; (**d**) in the X direction in the case of machining with a T02 cutting tool; (**e**) in the Z direction in the case of machining with the cutting tool T03; (**f**) in the X direction in the case of machining with the cutting tool T03.

In many situations, the vibration analysis using only the FFT method does not allow a proper analysis of the vibration phenomenon and, thus, in the next stage of experimental research a vibration analysis was performed using the Short-Time Fourier-Transformation (STFT) method. This method of analysis allows a clearer highlighting of the vibrations that occur throughout the processing process, because it also takes into account the time-based analysis of vibrations. The STFT method allows the tracing of some spectrograms, Figure 8, and from the analysis of the spectrograms information can be obtained on how the vibrations evolve over time. Vibration analysis performed only on the basis of stationary signals in a certain frequency range is not enough and, therefore, it is always necessary to use the STFT method, which is a dynamic method of analysis. Thus, the STFT analysis also allows an analysis of the transient signals, which is not valid when using the FFT analysis. STFT analysis is based on Discrete Fourier Transform (DFT) which provides information on the frequency and phase components of a section of a time-dependent signal. Optimization of the results obtained from the analysis of the vibration signal is possible if a high sampling frequency of the time signal is achieved and an appropriate resolution is used as a function of time and frequency [29,30].

The superimposed window technique is an optimal variant that can be used to establish the high frequency resolution in a time segment that can be used to trace vibration spectrograms, and this is justified by the fact that the analyzed machining process involves a movement of rotation of the part. Time segments can be considered quasi-stationary, and this was made possible by considering narrow enough time segments.

Following the STFT analysis, the vibration spectrograms were drawn in case the tool T01 (Figure 8a—direction Z; Figure 8b—direction X) is used in the processing, if the tool T02 is used in the processing (Figure 8c—direction Z; Figure 8d—X direction), respectively, for the case when the tool T03 is used (Figure 8e—Z direction; Figure 8f—X direction). 

All spectrograms shown in Figure 8 were analyzed, taking into account those mentioned above, in the frequency range 10–10,000 Hz. The analysis of the spectrograms allows the observation of the amplitude of the vibrations at certain moments of time and at a certain frequency. Thus, the use of vibration spectrograms allows a more complex analysis of the vibration phenomenon that occurs during the machining process of aluminum alloy.

The analysis of vibrations using spectrograms allowed the formulation of the following conclusions:

− the analysis of the Z-direction spectrograms showed that, when using the T01 tool, a maximum amplitude of vibrations is obtained at the beginning of the machining process, and this can be explained by the phenomena accompanying the cutting process at the tool entry into the cut. Additionally, the maximum value of the vibration amplitude was 6 m/s^2^. It should be noted that when using the smart tool T02 the vibration amplitude in the range up to 5000 Hz is approximately zero or very small. Thus, considering the analyzed frequency range, 10–10,000 Hz, it can be said that the use of the smart tool T02 determines the almost entire elimination of vibrations. If the T03 smart tool was used, the vibration amplitude has approximately the same values as in the case of the T01 tool, with a substantial increase in the vibration amplitude at very high frequencies. This demonstrates that the use of two spring washers is not indicated as it causes a decrease in the stiffness of the active part of the cutting tool, and this can increase the amplitude of the vibrations;
− although the magnitude of the cutting force acting in the X direction is much smaller than that acting in the Z direction, a STFT analysis was also performed and, in this case, the specific spectrograms were drawn, Figure 8b,d,f. The analysis of the spectrograms showed that the maximum values of the vibration amplitude are higher in the X direction than in the Z direction. This is explained by the fact that, although the cutting force is lower in the X direction compared to the one in the Z direction, however, the frictional force that causes the vibrations to occur is much higher on the face of the tool in the X direction. Thus, the use of the smart tool can allow to obtain an optimal functional geometry of the tool, especially in terms of the functional seating angle of the α_Fe_ tool. If the tool was used in machining in the classic construction version T01, a maximum value of the vibration amplitude of approximately 6.9 m/s^2^ was obtained. The use of the first type of T02 smart tool allowed a decrease in amplitude in the range of interest 10–10,000 Hz to a value of about 0.8 m/s^2^. Thus, it was demonstrated that the use of the T02 tool allows a considerable reduction in vibration amplitude by about 8 times. However, the use of the T02 tool is not recommended for the frequency value range between 5800 and 6000 Hz, when the amplitude of vibrations increases greatly up to 48 m/s^2^. The use of the T03 smart tool did not allow to obtain the best results in terms of vibration amplitude because vibrations have maximum values in the range of interest 10–10,000 Hz after which the vibration amplitude decreases showing an increase in amplitude value at a frequency value of 5000 Hz. This behavior of the T03 tool demonstrates that a smart tool must be constructively optimized given the design of a spring system arranged under the removable plate adapted to the conditions in which the cutting process takes place. It can also be concluded that the smart tool T02 has the best behavior in terms of vibration in the processing conditions of aluminum alloy A2024-T351.

Thus, the research confirmed that the analysis of vibration spectrograms allows to obtain useful information that can be used to design different smart tools based on the construction system presented in the research, and the use of such a smart tool allows reducing vibration amplitudes with positive effects on the quality of the surfaces and the precision of the machined parts [31].

The analysis of the vibrations that appeared during cutting by both the FFT and the STFT method allowed a very good observation of the vibration phenomenon, but, in practice, how the vibration parameters change over time must be known and especially what is the value of acceleration vibration. Under these conditions, in the next stage of experimental research, signal analysis was considered important, which gives information on the dependence of acceleration vibration as a function of time. Signal analysis was performed for all three tools (T01, T02, T03). This analysis of vibrations as a function of time allowed the monitoring of vibration levels at certain times. In the case of this signal analysis values were recorded for acceleration vibration which was measured in m/s^2^. The signal analysis requires a report on the acceptable limits of the operating vibrations of the system used for cutting, but these limits can be predefined by setting values with reference to certain standards. Thus, in this research, reference was made to an acceptable acceleration vibration limit of 40 m/s^2^ taking into account the recommendations in [32,33].

**Figure 9:**
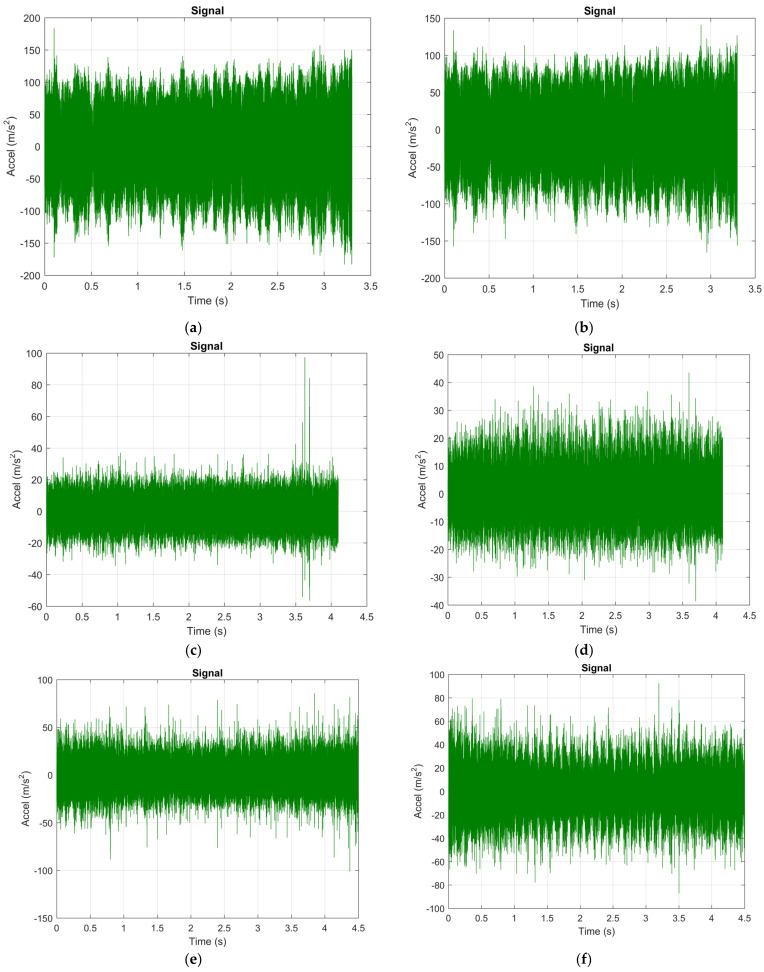
Acceleration of vibrations recorded during machining: (**a**) in the Z direction in the case of machining with cutting tool T01; (**b**) in the Y direction in the case of machining with a T01 cutting tool; (**c**) in the Z direction in the case of machining with a cutting tool T02; (**d**) in the Y direction in the case of machining with a cutting tool in variant T02; (**e**) in the Z direction in the case of machining with a T03 cutting tool; (**f**) in the Y direction in the case of machining with a T03 cutting tool.

The acceleration vibration signals obtained along the two directions Z and X, respectively, are presented in Figure 9 as follows: for the case when the tool T01 is used in the processing (Figure 9a—direction Z; Figure 9b—direction X), if, in machining, the tool T02 is used (Figure 9c—direction Z; Figure 9d—direction X), respectively, for the case when the tool T03 is used (Figure 9e—direction Z; Figure 9f—direction X).

From the signal analysis performed it was observed that the use of the tool T01 for both directions Z and X, respectively (Figure 9a,b), does not allow obtaining a value for acceleration vibration that falls within the imposed limit of 40 m/s^2^ and has an average value of about 100 m/s^2^. Thus, it is demonstrated that the use in the processing of a cutting tool in the classic construction variant (T01), determines the appearance of very high vibrations in the technological system. The replacement of tool T01 with the other smart tools T02 and T03, respectively, allowed a considerable reduction of acceleration vibration. Thus, the lowest values for acceleration vibration were obtained when using the tool T02 (Figure 9c,d), so that the average value of acceleration for this tool was in the range 18–20 m/s^2^, i.e., even lower than less than half of the maximum permissible value. In the case of using the tool T03, average values for acceleration vibration were obtained located below the maximum allowed value (Figure 9e,f). Thus, it is specified that, although in the cases of FFT and STFT analyses the tool T03 did not allow to obtain promising results, nevertheless, this type of tool can ensure a stability of the machining process from the point of view of vibrations.

The results obtained and presented in Figure 9 confirm that the geometry of the cutting tool has a very large influence on the vibrations that occur during the cutting process [34]. Thus, an improper geometry of the cutting tool can cause a substantial increase in the frictional forces that appear on the seating surface and the release of the tool, with negative effects on the acceleration vibration values. The use of a T02 cutting tool for machining can ensure optimum functional geometry during the machining process. Research has also shown that the presence of an unsuitable elastic element in the structure of the cutting tool, as in the case of the T03 tool, can cause an amplification of the vibration phenomenon caused by the decrease in tool rigidity, although such a constructive tool allows ensuring optimal functional geometry. In these conditions, it can be concluded that the constructive variant of smart tool T02 is a solution that can allow the considerable reduction of vibrations that occur during the cutting process. Thus, by using the T02 tool, a considerable improvement of the precision and quality of the processed surfaces can be obtained.

### 3.2. Results Obtained in the Analysis of the Own Modes of Vibrations Using FEM

**Figure 10:**
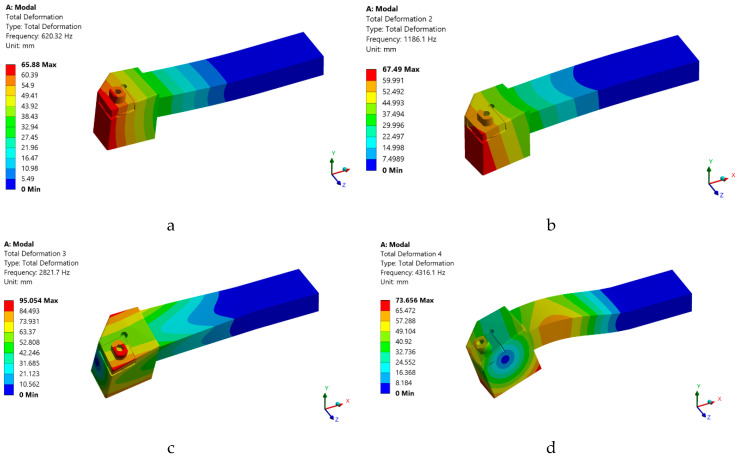

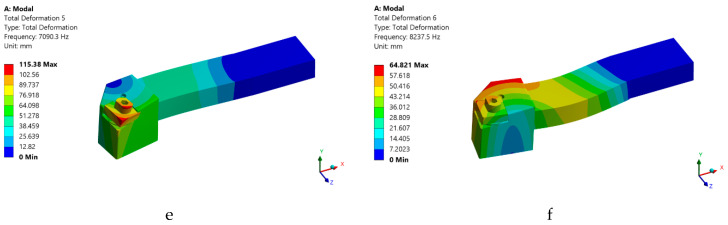
Own modes of vibration obtained by using FEM for the smart tool T02: (**a**) first own mode of vibration for a frequency of 620.3 Hz; (**b**) the second proper vibration mode for a frequency of 1186.1 Hz; (**c**) the third proper vibration mode for a frequency of 2821.7 Hz; (**d**) the fourth proper vibration mode for a frequency of 4316.1 Hz; (**e**) the fifth proper vibration mode for a frequency of 7090.3 Hz; (**f**) the sixth proper mode of vibration for a frequency of 8237.5 Hz.

At this stage of the research, a modal analysis was performed for the T02 tool to highlight the dynamic behavior of this constructive variant of tool. This modal analysis was performed only by using the T02 smart tool, for which the best results were obtained in the experimental research. By applying FEM, for tool T02, six own modes of vibration were obtained, Figure 10. From the analysis of the results obtained in the modeling it was observed that the first own modes of vibration (620.3 Hz) corresponds to a speed of 37,219 rpm, well above the working speed at 800 rpm. Consequently, there is no risk of resonance.

The results obtained by FEM modeling of the own numerical vibration mode showed that the use of the T02 tool, with self-adaptive geometry, allows the significant increase of the speed that determines the appearance of the first level of the own vibration mode. Thus, this type of smart tool can be used successfully even when the speed is very high without the danger of the resonance phenomenon. However, when the speed of the part is very high, there is a danger of an increase in vibration acceleration. In these conditions, it is demonstrated that the use of a smart tool type T02 can allow to obtain very good results in cutting and in the conditions of processing regimes that require high speeds of the part.

### 3.3. The Influence of the Use of Smart Tools on the Roughness of Surfaces Machined through Cutting

The roughness of the surfaces has a significant impact on the functionality of the parts and can influence the wear resistance, fatigue resistance, lubrication and friction, and optical properties. Thus, it is very important to establish the processing conditions in order to obtain the appropriate roughness [30]. In order to meet these requirements, the geometry of the cutting tool and the dynamic phenomena accompanying a cutting process must be taken into account. In many cases, an empirical parametric modeling of the surface roughness is required which can be performed considering the connection between the parameters of the cutting process (cutting depth a_p_, part speed and feed f) and the geometry of the cutting tool (rake angle γ, clearance angle α, etc.). As a conventional manufacturing process, turning technology has its unique characteristics. In addition to the parameters of the cutting process, for the analysis of surface roughness, certain dynamic aspects of the process must also be taken into account. Influencing factors in relation to the machine tool can be divided into kinematic aspects and dynamic aspects [35].

Thus, especially the vibration between the tool and the workpiece has an important role in forming the surface roughness. Very often there is a connection between the amplitude of the vibration between the part and the roughness profile. Under these conditions, the research aimed to create a smart tool to control the vibration between the tool and the part. As the vibration effect is complicated and difficult to integrate into the theoretical formulas of surface roughness, it was necessary to apply an indirect method of surface roughness analysis. In the case of the indirect method, a surface topography model is first established taking into account vibration. Tool tip vibrations can be controlled by using a smart tool during machining, and controlling tool vibration and feed rate can have very good effects on surface roughness. At the same time, previous studies have shown that a control of vibrations in the two directions Z and X, respectively, can have a very good effect on the surface roughness [36,37].

Given the specified research, the focus was on the analysis of tool vibrations that establish a correlation between them and the surface roughness obtained. The roughness analysis was performed both for the tool in the classic construction version T01 and for the two tools in the smart construction version T02 and T03, respectively. This analysis was necessary because the vibrations that occur during cutting substantially influence the roughness of the machined surface. The design of the smart tools was aimed at ensuring during the machining process an optimal functional geometry for the cutting tool that would allow the control of the vibrations of the tip of the cutting tool so as to obtain the best possible surface roughness.

For a good interpretation of the experimental results when determining the roughness, the measurements were repeated 10 times at the same distance from the end of the turned bar, for each type of tool used. For a correct evaluation, the piece was indexed by approximately 36° between two measurements. An analysis was also performed to eliminate outliers, as these may lead to disproportionate results of statistical analyses. Boxplot analysis, for the elimination of outliers was performed with the help of the Minitab program, aberrant results not were identified. Additionally, with this program the normality of the data distribution was verified, calculating mean, median value, and standard deviation. The test used to verify normality was derived from the Kolmogorov–Smirnov test, Andreson–Darling test, respectively, due to the fact that this test is one of the most sensitive tests used to verify the normal distribution.

**Figure 11:**
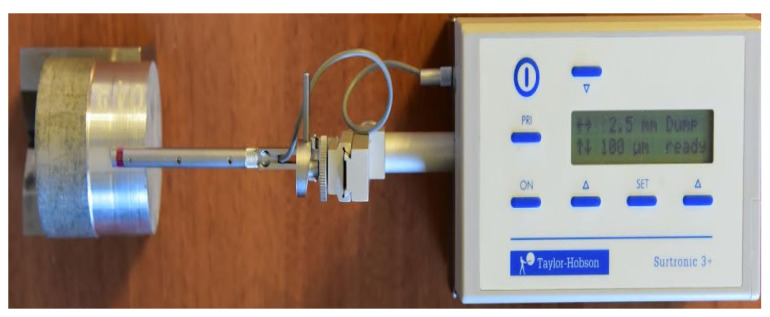
Images with surface roughness obtained by cutting machining.

In order to ensure the adequacy of the experimental research, 10 samples were processed under the same conditions for which the roughness was measured. The roughness of the obtained surfaces was measured for each type of tool used for processing (T01, T02, T03). The roughness values measured for the 10 samples processed with the three types of tools are shown in Table 3. Additionally, an image of the roughness measurement process is shown in Figure 11, and the sample shown in the figure is sample1, processed with tool T02, which had the lowest roughness value.

**Figure 12:**
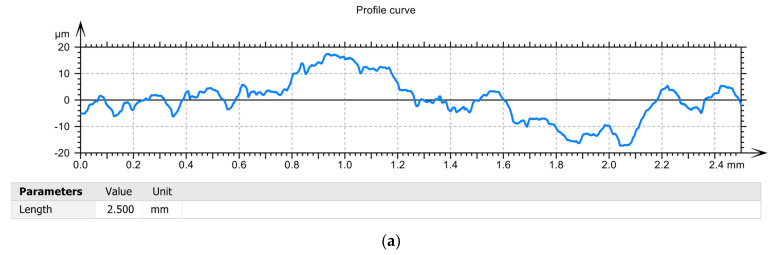

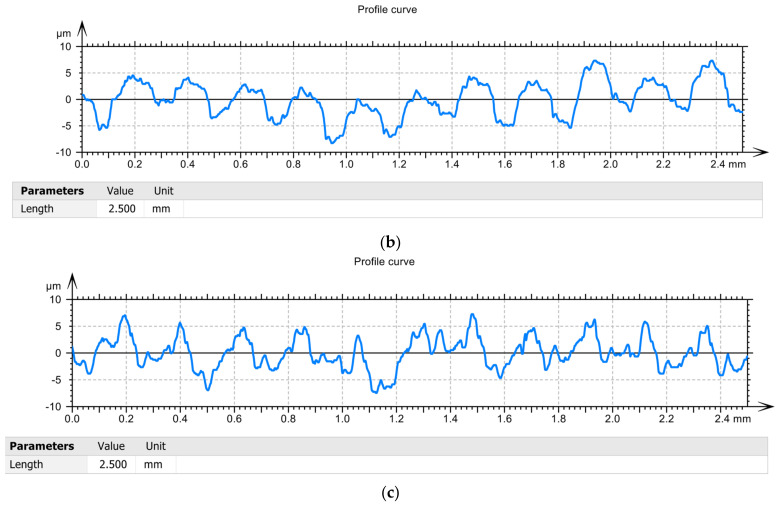
Profile curve: (**a**) in the case of machining with a T01 cutting tool; (**b**) in case of machining with T02 cutting tool; (**c**) in case of machining with cutting tool T03.

After measuring the roughness of the parts’ surfaces, different values were found in the case of using the three types of tools, Table 3 and Figure 10, respectively. Thus, the highest values of roughness were obtained in the case of using the T02 smart tool. Additionally, the lowest roughness was obtained in the case of the first sample processed with tool T02, and this can be explained by the fact that at the beginning of processing the removable plate was new, as well as the spring washer. At the same time, these roughness values demonstrate that there is a correlation between them and the values of the parameters obtained in the vibration analysis stage. From Figure 12, it is observed that the maximum values for curved profiles were obtained when using the tool T01 (approximately 20 µm), Figure 12a, and the lowest values were obtained when using the tool T02 (approximately 5 µm), Figure 12b. In case of using the tool T03 for curved profiles, maximum values of approximately 7 μm were obtained, Figure 12c, which are lower than the situation of using T01, but higher than the situation of using T02. This demonstrates that the way the elastic system used to make smart tools has a great influence on the control of the vibrations of the tool tip, with effects on the roughness of the workpiece surface. Thus, the use of an elastic system made of two overlapping spring washers (T03—Figure 1c) is not optimal, because a decrease in the rigidity of the technological system is obtained and the best conditions regarding the optimal functional geometry of the tool are not ensured.

**Figure 13:**
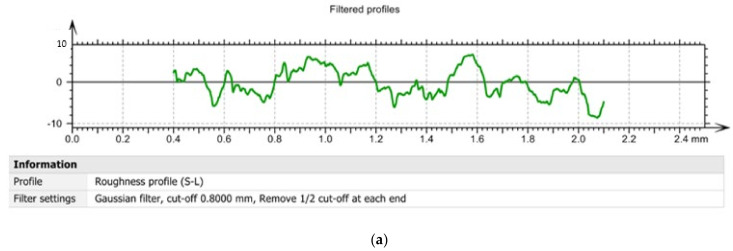

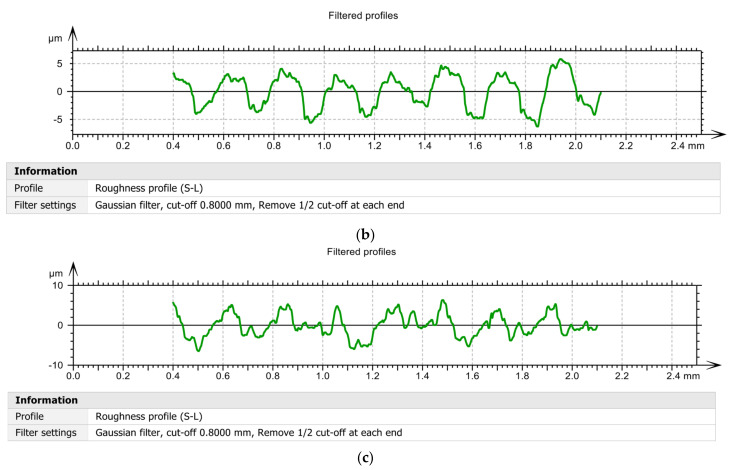
Filtered profiles: (**a**) in the case of machining with a T01 cutting tool; (**b**) in case of machining with T02 cutting tool; (**c**) in case of machining with cutting tool T03.

By processing the experimental data, a series of curves related to the filtered profiles were drawn, Figure 13, which highlights the advantage of using the T02 tool for the roughness of the processed surfaces. The use of filtered profile curves allows the elimination of those wavelengths located outside the band of interest. From the analysis of the values presented in Figure 13 it was observed that the highest values of the asperities from the filtered profiles were obtained when using the tool T01 (approximately 10 µm), while in the case of tool T02 and tool T03, values below 5 and 7 μm, respectively, were obtained. Thus, the values obtained by tracing filtered profiles confirm the results obtained by tracing curved profiles. It also confirms the direct link between the vibrations that occur during the machining process and the roughness of the machined surfaces [38,39].

**Figure 14:**
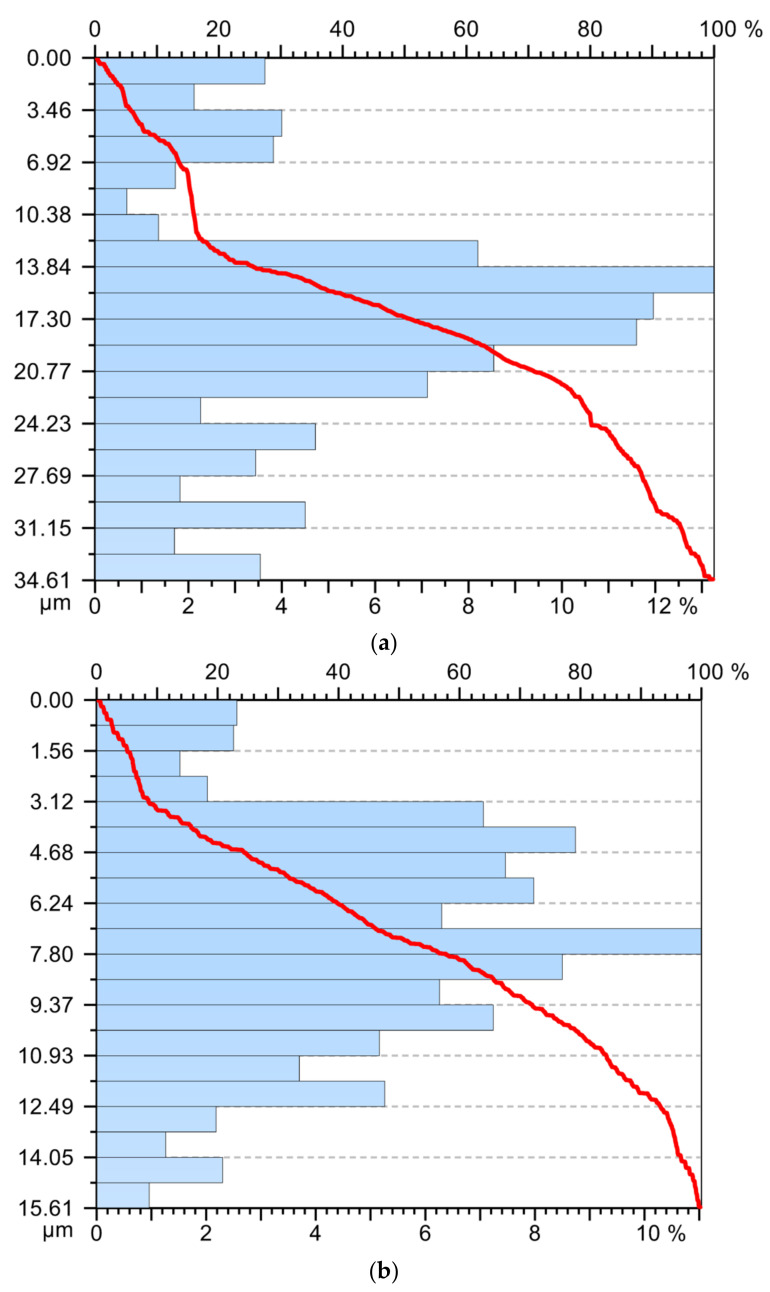

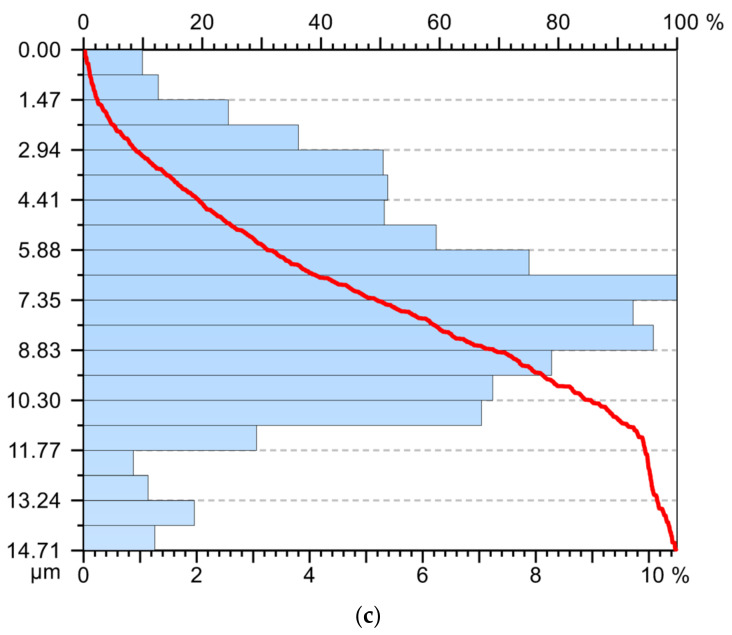
Abbott–Firestone curve: (**a**) in the case of machining with a T01 cutting tool; (**b**) in case of machining with T02 cutting tool; (**c**) in case of machining with cutting tool T03.

For the 10 samples, the experimental data obtained were processed, and the graphs presented in Figure 14 were drawn. From the analysis of the evolution of the curves highlighted in Figure 14 it was observed that the smart tools T02 and T03 offer the best stability in terms of roughness, while tool T01 does not meet this requirement. This can be explained by the fact that tool T01 does not have the possibility of vibration control and does not allow to ensure a proper functional geometry.

Although the results obtained in terms of surface roughness have been very promising, there may still be a number of aspects that can be optimized, such as, for example, those related to the characteristics of the damping system arranged under the removable plate. Under these conditions, numerical simulation methods can be used for the constructive optimization of the elastic system, such as finite element simulation and molecular dynamics simulation. Tool wear must also be considered, which inevitably occurs during turning and increases with the machining process. At the same time, it would be necessary to achieve a correlation between the surface roughness and other parameters such as cutting temperature, cutting tool vibration, tool wear, and durability.

To monitor the vibrations that occur during turning, some research has proposed smart tools with fiber Bragg grating (FBG) sensors. This type of smart tool offers a number of advantages such as immunity to electromagnetic interference, resistance to explosion, the ability to measure multiple shear forces [40,41]. However, these types of smart tools are not possible to use in the processing of aluminum alloys because they are sensitive to the use of coolants.

Additionally, smart tools were made using shear thickening fluid (STF), which, when turned, allowed a damping of vibrations and an improvement of the surface quality of the workpieces. However, this constructive form of smart tool implies a decrease in the rigidity of the tool due to the holes processed in its body [42,43].

All these smart tools presented in recent research have certain limits. As a result, the intelligent tool used in research differs from them in that it allows monitoring, but also a permanent adjustment of forces and vibrations, ensuring optimal conditions for processing aluminum alloys. The smart tool also allows an instantaneous continuous adjustment of the parameters without the need for certain response times. The design of the smart tool and the results obtained have shown that such a tool falls into the category of smart tools because it allows not only a monitoring of cutting conditions but also a permanent correction of the parameters of the cutting process to ensure both a reduction of the vibrations and a roughness as low as possible.

## 4. Conclusions

The research carried out allowed the design and production of two smart tools, T02 and T03, respectively, which can be used in optimal conditions for processing aluminum alloys. By using these types of smart tools, a reduction in tool tip vibrations was achieved with positive effects on the roughness of the machined surfaces. Obtaining a smart tool is possible by optimizing the elastic system arranged under the removable plate. Testing of smart tools in the process of turning aluminum alloys has shown that:

▪ the use of the smart tool T02 determines the almost entire elimination of vibrations, and if a smart tool T03 with a non-optimized elastic system was used, a substantial reduction of the vibration amplitude was not achieved, having approximately the same values as in the case of the tool made in classic constructive variant;
▪ the use of a system consisting of two spring washers is not indicated as it causes a decrease in the stiffness of the active part of the cutting tool, and this may increase the amplitude of the vibrations;
▪ vibration analysis by the FFT and STFT method, respectively, allowed an in-depth analysis of the vibration amplitude for a frequency of up to 10,000 Hz;
▪ FEM modeling of the own numerical vibration mode of the tools used in the machining, demonstrated that the use of smart tools allows to greatly increase the speed of the part at which the first proper vibration mode appears. Thus, this type of smart tool can be used successfully even when the speed is very high without the danger of the occurrence of the resonance phenomenon. Additionally, from the analysis of the results obtained in the case of FEM application, it was observed that the first own vibration mode (620.3 Hz), corresponds to a speed of 37,219 rpm, well above the working speed of 800 rpm;
▪ the use of smart tools allows to obtain very low roughness values compared to the roughness values obtained compared to the roughness obtained when using tools in the classic construction version;
▪ the results obtained in terms of surface roughness were very promising, but there may still be a number of aspects that can be optimized, such as, for example, those related to the characteristics of the damping system arranged under the removable plate;
▪ the research results, using the proposed tool, showed that it can be considered a smart tool that allows an instantaneous continuous adjustment of the parameters of the turning process, without the need for certain response times, as in the case of other types of smart tools.

The present research has shown the advantages that smart tools offer in the processing of aluminum alloys, both technically and economically. Future research will aim to optimize the constructive shape of smart tools, so that they can be used successfully in the processing of other materials.

## Figures and Tables

**Table 1:** Chemical composition of aluminum alloy A2024-T351, %.

Al	Cr	Cu	Fe	Mg	Mn	Si	Ti	Zn
90.7–94.7	max. 0.1	3.8–4.9	max. 0.5	1.2–1.8	0.3–0.9	max. 0.5	max. 0.1	max. 0.25

**Table 2:** Mechanical properties of aluminum alloy A2024-T351.

Properties	Value	Comments
Hardness, Brinell	120	AA; Typical; 500 g load; 10 mm ball
Ultimate Tensile Strength	469 MPa	AA; Typical
Tensile Yield Strength	324 MPa	AA; Typical
Elongation at Break	19%	AA; Typical; 1/2 in. (12.7 mm) Diameter
Modulus of Elasticity	73.1 GPa	AA; Typical; Average of tension and compression. Compression modulus is about 2% greater than tensile modulus.
Ultimate Bearing Strength	814 MPa	Edge distance/pin diameter = 2.0
Bearing Yield Strength	441 MPa	Edge distance/pin diameter = 2.0
Poisson’s Ratio	0.33	
Fatigue Strength	138 MPa	AA; 500,000,000 cycles completely reversed stress; RR Moore machine/specimen
Shear Modulus	28 GPa	
Shear Strength	283 MPa	AA; Typical

**Table 3:** The measured roughness values for the 10 samples, Ra, µm.

The Sample Number	In Case of Machining Performed with Tool T01	In Case of Machining Performed with Tool T02	In Case of Machining Performed with Tool T03
1	2.717397	1.448305	2.506057
2	2.172855	1.468041	2.471939
3	2.477422	1.526573	2.389087
4	2.646282	1.581081	2.262302
5	2.464776	1.520499	2.162325
6	2.626595	1.598336	2.137241
7	2.237116	1.646847	2.388526
8	2.263172	1.688449	2.270452
9	2.173006	1.693019	2.347753
10	2.281989	1.700883	2.406131
Mean	2.406061	1.606203	2.232181
St Dev	0.206791	0.084961	0.120591
Cvariation	8.594601	2.259944	5.17075
Median	2.373382	1.598728	2.36814
*p*-value	0.197	0.94	0.504

## Data Availability

Not applicable.
